# The Current Challenges in Diagnosing and Treating Malignant Priapism: A Comprehensive Review

**DOI:** 10.5152/tud.2023.23088

**Published:** 2023-11-01

**Authors:** I Wayan Yudiana, Ronald Sugianto, Putu Eka Dianti Putri, Ni Putu Ekawati, Gede Wirya Kusuma Duarsa

**Affiliations:** 1 Department of Urology, Universitas Udayana, Prof. Dr. I.G.N.G Ngoerah General Hospital, Faculty of Medicine, Bali, Indonesia; 2 Universitas Udayana, Medical Doctor Study Program, Faculty of Medicine, Bali, Indonesia; 3 Universitas Hang Tuah, Medical Doctor Study Program, Faculty of Medicine, East Java, Indonesia; 4 Department of Anatomical Pathology, Universitas Udayana, Prof. Dr. I.G.N.G Ngoerah General Hospital, Faculty of Medicine,Bali, Indonesia

**Keywords:** Penile metastases, priapism, diagnostic, prognostic, radiotherapy, surgical treatment

## Abstract

Malignant priapism (MP) is defined as a condition of persistent erection of the penis without sexual stimulation due to the neoplastic process of the cavernous sinus and the efferent veins. The effectiveness of established therapeutic recommendations in priapism was ineffective in MP. Modalities of therapy for MP varied from medication treatment, nonsurgical treatment, surgical treatment, and radiotherapy. Despite aggressive surgical management combined with radiation therapy, chemotherapy, or targeted therapy, the survival rate remains low. Therefore, the treatment is usually palliative, focusing on the patient’s quality of life improvement and symptom relief.

**Main Points**

- Malignant priapism is defined as priapism due to the neoplastic process, which arises mainly from the genitourinary tract in approximately 63%- 70% of cases.
- The most popular hypotheses are through 3 different etiopathogeneses: direct neoplastic cell infiltration into the corpora cavernosal, penile metastases, and increasing viscosity and clotting of blood in the subtunicalropical emissary vein by the neoplastic cells.
- The diagnosis tools include anamnesis, physical examination, laboratory examination, tumor markers, bone marrow biopsy, histological examination of the penile tissue, and imaging, including penile ultrasonography, computed tomography (CT) scan, and magnetic resonance imaging (MRI); The first management of MP should be focused on the emergency of priapism and relieving the pain, while the further management is to limit the progression of the disease, such as radiotherapy, chemotherapy, and brachytherapy.

## Introduction

Priapism is a prolonged erection, full or partial, for more than 4 hours without sexual stimulation.^1-3^ The etiologies of priapism include hematological disorders such as sickle cell anemia, neurological damage, trauma, infection, malignancy, erectile dysfunction drugs, such as papaverine and alprostadil, and metabolic disturbances. The clinical manifestation of priapism can be divided into 3 subtypes: ischemic priapism (IP), nonischemic priapism (NIP), and recurrent priapism (RP).^4,5^ Priapism caused by invasion or metastasis from a neoplasm process, a rare cause, is called malignant priapism (MP).^3^ Malignant priapism was described first by Peacock^6^ in 1938. Malignant priapism is one of the most frequent presentations of penile metastases, which arises mainly from the genitourinary tract in approximately 63%-70% of cases followed by the gastrointestinal system in about 19%.^7,8 ^The most common primary lesion was from the prostate, bladder, and recto-sigmoid and rarely from the kidney, testes, lung, stomach, bone, ureter, hepato-biliary, and urethra.^9^ The other presentations of secondary malignancy of the penis include penile masses, indurated nodules, ulcers, and skin lesions.^4^ The studies discussing about penile metastasizes were still lack yet ^3^

According to European Association of Urology guidelines on Priapism 2022, the effectiveness of the conventional therapeutic recommendations in priapism was low in MP.^10^ Therefore, our review study presents the current treatment modalities and the prognosis of MP patients.

## Material and Methods

The literature searching was conducted with the medical subject heading terms “malignant priapism” and “management” in electronic databases, including PubMed/MEDLINE, Cochrane Library, EMBASE, and Scopus, and publicized studies from Januarry 2003 to May 2023 following Cochrane Highly Sensitive Search Strategy.^11^ The literature was screened by title and abstract to find the eligible studies as our literature. There are no exclusion criteria for the study design, but randomized control trials (RCT) studies, meta-analyses, and observational studies are more prioritized for review.

## Discussion

Penile metastases are a rare presentation and are associated with severe illness. One of the presentation of penile metastases is priapism, considered as MP. The spreading of malignant cells was hypothesized through venous or lymphatic occlusion that caused impaired outflow and enhanced the neoplastic spreading. .^12^

### Classification

Depending on the pathophysiological cause of priapism, the clinical manifestation of priapism can be divided into 3 subtypes: IP, NIP, and RP.^2,10^ Around 95% of all incidences of priapism are IP, and most cases are iatrogenic, which are caused by the use, misuse, and recreational use of ED medications, specifically intracavernosal injection medications, whereas NIP is typically associated with perineal/pelvic trauma.^13,14^ The pathogenesis of IP is complex and not fully understood, which hypothesized that neurotransmitter dysregulation with vascular and neuronal factors affects the smooth muscle of the corpora cavernosal. In IP manifestation, the penile tissues can suffer progressive hypoxia, hypercapnia, and acidosis, leading to smooth muscle dysfunction, ischemia, infarction, and dense fibrosis as complications., while NIP is pain free with a partially tumescent penis associated with perineal or penile trauma that results in an arteriovenous fistula in the corpora cavernosal.^2,15-17^ Recurrent or stuttering priapism is a recurrent self-limiting episode of priapism which is frequently observed in patients with hematological dyscrasias.^2,15-18^

Even though malignancy as an etiology of priapism is rare, it should be considered the etiology in individuals without any other risk factors.^2^ Malignant priapism can be caused by direct invasion of the primary penile tumor or invasion from the secondary metastatic tumor.^14^

### Etiology

Many theories have been proposed to explain how the original tumor, far from the penis, reached the penis, but the main mechanism remains unknown. However, the most popular hypotheses are through 3 different etiopathogeneses: direct neoplastic cell infiltration into the corpora cavernosal, penile metastases, and increasing viscosity and clotting of blood in the subtunical emissary vein by the neoplastic cells.^19^ The most popular hypothesis was that the neoplastic cells spread to the penis tissue through antegrade arterial dissemination, retrograde Batson plexus flows, lymphatic route, or iatrogenic, which is uncommon.^7,20^ The second theory was that malignant cells are responsible for increasing leukocytosis and kostasis, which lead to a hyperviscosity state and encourage the development of microthrombi that might block the cavernosal sinusoids and the efferent veins, resulting in cavernosal congestion. This theory also promotes the enhancing cytokine and adhesion molecule production, engaging endothelial cells and leading to a condition called cellular sequestration that might result in microvascular blockage. Another hypothesis was that neoplastic cells invade sacral nerves or the central nervous system, which would significantly interrupt and interfere the erection pathways. However, the main etiology is still unknown and debatable to many other theories.^14,21^

### Diagnosis

Because of the rare incidence and features similar to other priapisms, MP is often initially misdiagnosed and causes delayed treatment. Therefore, anamnesis of priapism should be emphasized taking into account several important factors, including the duration of the priapism, the intensity of the pain, the presence of prior priapism episodes, the state of erectile function before the priapism episode, and histories of the patient.^14^ Malignancy should be considered as an underlying cause, especially in elderly patients in the sixth decade of life, with a family or previous history of malignancies without any prior medical history. In patients without a malignancy history, the specific symptoms that lead to suspicions of malignancy should be investigated.^20,22,23^

Physical examination is also crucial to distinguish IP, which increases cavernosal rigidity accompanied by local pain, while NIP characteristics are engorged corpus cavernosum without rigidity and pain.^10^ Observation of the skin lesion and nodule in the penis is also important to suggest the suspicious malignancy condition that explains priapism onset. For patients highly suspected of malignancy without a malignancy history, the diagnosis of neoplasm should be done simultaneously with priapism treatment. The classic symptoms of malignancy, which must be aware of, were cachexia, wasting, and organ-related neoplasm symptoms.^14,21^

**Figure 1.**
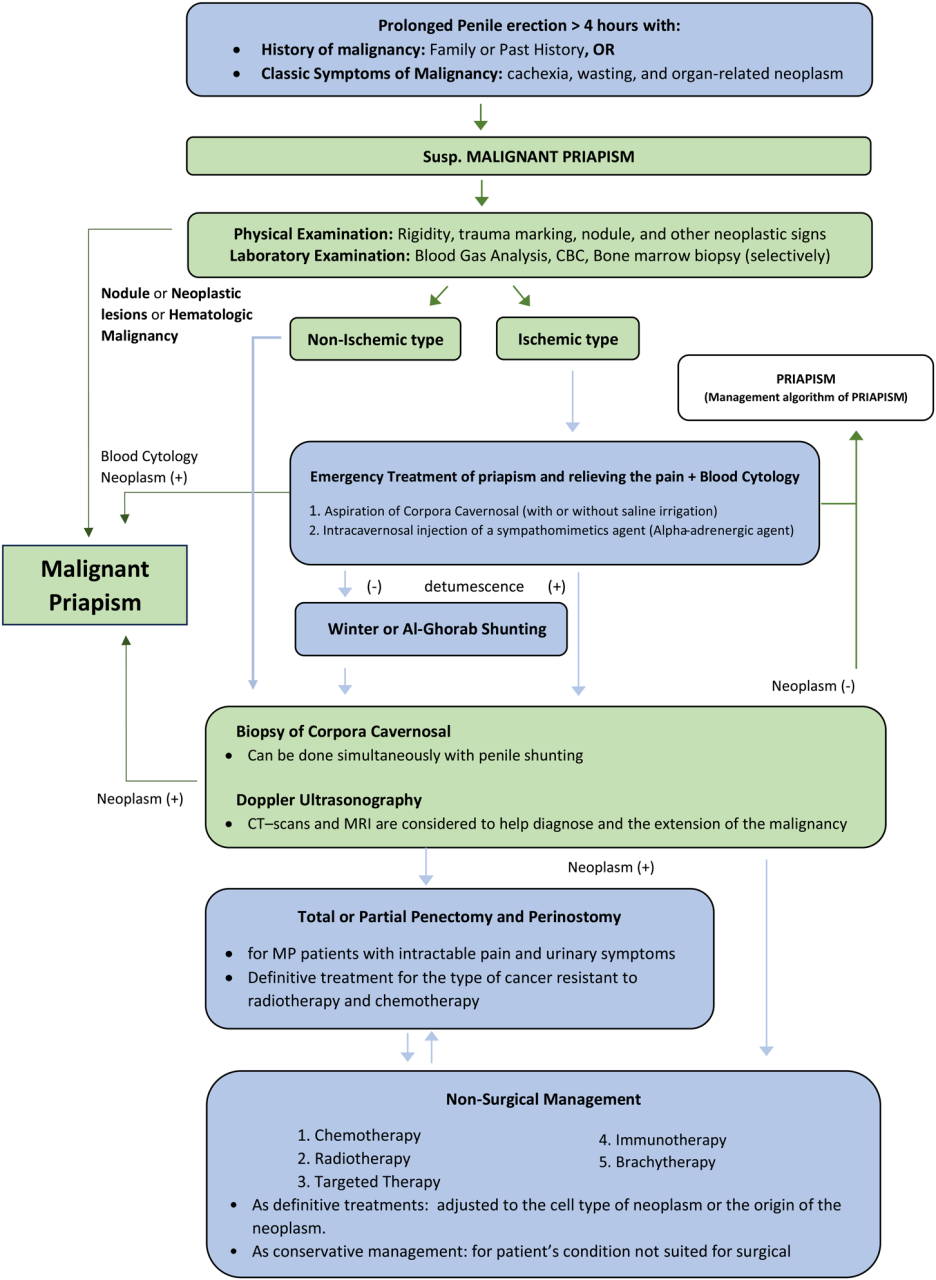
Diagnostic and management of malignant priapism are performed simultaneously. The green color indicates the diagnostic flowchart, while the blue color indicates the management flowchart. CBC, complete blood count; CT, computed tomography; MP, malignant priapism; MRI, magnetic resonance imaging; Susp, suspension.

Further investigations of MP include laboratory examination, tumor markers, bone marrow biopsy, histological examination of the penile tissue, and imaging, including penile ultrasonography, computed tomography (CT) scan, and magnetic resonance imaging (MRI). The essential laboratory examinations were complete blood count, blood film, and white blood cell count with differential count. This examination can rule out the hematologic abnormality or hematologic malignancy commonly presented as an underlying cause of MP. Bone marrow biopsies were performed selectively, only for the inconclusive laboratory examination.^21^ Penile ultrasonography is a fast and reliable modality to detect penile metastatic lesions and differentiated high- and low-flow priapism. The additional contrast-enhanced ultrasound gives more advantages that enable real-time imaging to determine the vascularity of metastases.^8^ Computed tomography scans and MRI are considered noninvasive modalities that help diagnose and determine the extension of the malignancy.^25^ Penile MRI can provide high-quality images of the penile tissue and viability of the cavernosal smooth muscle fibers, which helps identify bleeding and thrombosis inside the corpora by T1-weighted sequence MRI. ^14^ However, the definitive diagnosis and differentiating penile metastases from the neoplasm process are only made by biopsy or fine-needle aspiration and histopathological analysis. The workup diagnostic of MP is summarized in Figure 1.

### Management

The management of MP should be focused on the emergency of priapism and relieving the pain. Ischemic MP needs immediate treatment to reduce the penile compartment syndrome, relieve the pain, reestablish the blood flow to the penis, and decrease the risk of permanent erectile dysfunction. The first procedure to achieve detumescence is an aspiration of corporal cavernosal with or without cavernosal saline irrigation combined with fine-needle aspiration, which can be used for corporal blood glass analysis for IP and blood cytology diagnosis of MP.^3,21,24^ If the aspiration and irrigation of corpora cavernosal do not improve symptoms, the following intracavernosal injection of a sympathomimetics agent, preferably an alpha-adrenergic agent, can be given. However, the physician should monitor blood pressure and heart rate during the injection of alpha-adrenergic agent for evaluating the tachycardia and reflex bradycardia as discussed in American Urological Association (AUA) and American Society of Reproductive Medicine (ASRM) guidelines.^1,14,21^ In the case of conservative and nonsurgical treatments of MP failure, surgical intervention is recommended by making an iatrogenic fistula to drain deoxygenated blood from the corpora cavernosal through several techniques of penile shunting, such as winter shunting or Al-Ghorab shunting.^24^ During the penile shunting procedure, the surgeon should have cell biopsies of corpora cavernosal to be performed for a histopathology examination. More invasive shunts or prosthesis surgery were not recommended due to the high risk of cancer's dissemination. Therefore, after the initial management of priapism, MP patients should have multidisciplinary care for treatment management.^2,20,21^

Further management to limit the progression of the disease through radiotherapy, chemotherapy, and brachytherapy should be considered, even though some studies said it has no survival benefit. The treatment modalities were adjusted according to the cell type of neoplasm or the origin of the neoplasm. In renal cell carcinoma (RCC), radiotherapy can be a treatment modality that significantly improves MP outcomes.^29^ However, the guideline for optimal dose and fractionation in radiotherapy has not been published yet. High-dose palliative radiotherapy (40-50 Gy) is recommended due to the beneficial outcome for MP secondary to prostate, urothelial, and rectal carcinoma.^13,26-29^ The volumetric modulated arc therapy (VMAT), which is moderately hypo-fractionated high-dose radiotherapy, is preferred because of the simple patient setup and reducing patient stress. Specific in genitourinary or lower intestinal malignancies, which require high-dose radiotherapy and palliative pelvic radiotherapy, VMAT can minimize pelvic tissue damage.^29^ However, for the type of cancer resistant to radiotherapy and chemotherapy, persistent pain, and urine symptoms, surgical management, such as partial or complete penectomy, may be an effective therapy. Although total penectomy might cause psychological trauma and has limited survival benefits, it is pain free for the patient and improves the patient’s quality of life.^22,23,30^ The management of MP is summarized in Figure 1.

### Prognosis

Despite aggressive surgical management combined with radiation therapy, chemotherapy, or targeted therapy, the survival rate was not compromising. Since MP was first identified, the average duration between penile metastases and death has been between 1 week and 18 months, with an average of 6 months. No patients have lived beyond 18 months, and the survival rate of MP is often less than a year following diagnosis.^9,31^ The patients with rectal primary tumors, who developed MP, appear to have the longest survival time, over 9 years. Among the genitourinary primary tumors, prostatic adenocarcinoma has the longest survival time, up to 7 years.^9,32^ Moreover, the study showed that the 18-month mortality of MP was 64%, a mean of 7.57 months, while another study demonstrated that 60% of patients died within 6 months of treatment.^2,33^ In contrast, a cohort study conducted by Cocci et al^34^ showed a better cancer survival time (CaST) when MP was presented as the first symptom of metastases; the median CaST of MP from urogenital origin was 30 months, which is longer than other origins with a median of 15 months.^29,34^ Therefore, MP tends to have a poor prognosis, but it depends on the primary cancer.

Finally, the treatment modalities of MP, such as radiotherapy, systemic chemotherapy, local excision, penectomy, or a combination of these treatments, depend on the patient’s clinical conditions, even though the treatment is usually palliative, focusing on the patient’s quality of life improvement and symptom relief. 

## Conclusion

Malignant priapism is an uncommon type of priapism that spreads through direct neoplastic cells infiltration into the corpora cavernosal, penile metastases, and increasing viscosity and clotting of blood in the subtunical emissary vein by the neoplastic cells. Modalities of therapy for MP varied from medication treatment, nonsurgical treatment, surgical treatment, and radiotherapy. Despite aggressive combined treatment, the survival rate remains low and may worsen within months.
